# Supplementary material for: Are carbon emissions trading and green financial instruments synergistic? -Comprehensive quantitative research based on content analysis
Source: PLoS One. 2024 Mar 7;19(3):e0298601. doi: 10.1371/journal.pone.0298601 (PMC10919601; doi:10.1371/journal.pone.0298601)
Supplement: S1 Data — (ZIP) [file pone.0298601.s001.zip › Data/地级市Z_F工作报告.zip!/╣π╢1⁄2/╘╞╕í╩╨/└┤╫╘╣1⁄2╓┌║┼í╛┬φ┐╦╩2╛▌═°í┐_2006.docx]

各位代表：

我代表市人民政府向大会作政府工作报告，请予审议，并请政协委员和其他列席人员提出意见。

“十五”时期的工作回顾

“十五”时期是我市改革开放和现代化建设加快推进、经济建设和社会发展不断取得新成绩的五年。市政府在省委、省政府和市委的正确领导下，在市人民代表大会及其常委会和市政协的监督支持下，高举邓小平理论伟大旗帜，努力实践“三个代表”重要思想，树立和落实科学发展观，围绕建设山区经济强市的总目标，坚持以加快发展为主题，加大力度推进工业化、城镇化和农业产业化发展，积极实施“十项民心工程”，有效应对经济运行中种种不利因素，成功战胜非典型性肺炎、高致病性禽流感疫情及超百年一遇西江特大洪水等重大自然灾害，经济社会持续快速协调健康发展。经过全市上下的共同努力，顺利完成了市三届人大二次会议确定的2005年各项预期目标任务，圆满完成市二届人大三次会议确定的“十五”计划各项任务。

——经济保持平稳较快发展态势。按2004年经济普查的口径，2005年全市生产总值达到215.3亿元，比上年增长13.6%；五年年均增长8.7%，比“九五”时期提高3.7个百分点。按可比价计算，一、二、三次产业比例由2000年的39.8:29:31.2发展为36.1:33.3:30.6。工业总产值、农业总产值分别实现230.4亿元和119.66亿元，分别比上年增长27.1%和7.2%，五年年均分别增长11.1%和6%；社会消费品零售总额、固定资产投资总额分别实现59.63亿元和107.31亿元，分别比上年增长17.8%和27.8%，五年年均分别增长13.7%和26.6%。实现进出口总额7.33亿美元,其中出口总额4.31亿美元,分别比上年增长21.7%和23.1%，五年年均分别增长17.4%和16.3%。实际利用外资0.38亿美元，比上年增长77.7%，五年年均增长17.5%。地方财政一般预算收入9.33亿元，增长24.7%，五年年均增长20.6%。国税、地税分别实现9.94 亿元和8.65亿元,比上年增长21.4%和24.6%，五年年均分别增长17%和26.6%。金融机构各项存款余额213.66亿元，比上年增长14.8%，五年年均增长13.3%。城镇在岗职工工资总额21.22亿元，比上年增长20.4%，五年年均增长12%。农村居民人均年纯收入4227元,比上年增长5.6%，五年年均增长4.4%。

——农业和农村经济发展加快。2005年，全市粮食播种面积237.4万亩，总产量96.2万吨，连年超额完成省下达的任务。罗定市被评为我省唯一的全国粮食生产先进县。农业产业化经营组织发展到135个，比上年增加20个，其中农业龙头企业发展到82个，比上年增加10个；2005年农业产业化经营组织销售收入107.5亿元，带动农户19.5万户，户均增收4628元。劳务经济进一步发展，外出务工达到44.77万人，实现劳务年收入20.6亿元。建成了罗定粤西泷州农产品交易中心等农产品交易市场。五年来，水果、蔬菜、花卉、蚕桑、药材等经济作物和畜牧、水产养殖业有较大发展，涌现出“中国肉桂之乡”、“中国果品加工之乡”、“中国柑桔产业龙头县”和“中国无核黄皮之乡”等一批特色农业商品生产基地。共组织3次农业招商引资活动，累计签订项目59个，合同金额14亿元。

——工业呈现加快发展势头。加快发展资源开发和加工型工业，积极引进和发展新兴工业，采取有力措施化解煤、电、油、运对工业发展的制约，加大承接珠三角产业转移和招商引资力度，发展工业园区，组织项目建设，推动全市工业加快发展。一些科技含量较高的制药、电子信息产业产值比上年分别增长了64.3%和53.2%。五年来，省直管的硫化工和电力等企业规模扩大，县（市、区）的石材、水泥、不锈钢制品、纺织服装、机械、电池和农产品加工等产业继续加快发展，镇（街）工业发展步伐加快，涌现出一批工业专业镇。承接珠三角地区产业转移工作取得初步成效，与佛山市共建的都杨和新成产业转移工业园区已动工建设。“十五”期间，全市共引进各类项目630多个，计划投资总额140多亿元。其中2005年新上项目413个,计划投资总额70.4亿元。重大项目筹建、报批工作取得新进展，云浮电厂三期省已上报国家发改委，华润西江发电厂已列入省“十一五”规划，“西水南调”项目省已确定开展预可行性研究，粤泷电厂二期等重大项目前期工作进展较好，核电项目已与有关机构签订了合作开发框架协议，厂址已经国家电力规划设计院预评审通过。罗定市双东环保工业园、郁南县大湾环保工业基地和云安县六都工业园的规划建设已经启动。

——旅游、服务和物流等产业逐步发展。充分利用我市旅游资源加快旅游业发展，全市新建了市迎宾馆等一批宾馆酒店，开发了罗定龙湾、郁南古民居等生态旅游景点，进一步改造和完善了蟠龙洞、新兴龙山等风景区建设。2005年全市接待旅客人数205.62万人次，实现旅游总收入16.06亿元，比上年增长11.84%。城乡消费持续升温，商贸、物流持续畅旺。2005年全市新增在建商贸服务业项目55个，总投资11.6亿元。2005年全市汽车拥有量达3.4万辆，比“九五”期末增长58.1%；电话用户87万户，增长141.7%；邮政业务总量增长184%;互联网普及率大幅提高；货物吞吐量达到653.6万吨，增长251.4%。保险业、证券业稳健运行，中介业务稳步增长。

——发展空间不断拓宽。“十五”期间，我市抓住我国加入世贸组织的机遇，不断提高对外经济合作水平，在巩固港澳和东南亚市场的同时，积极拓展台湾、日本和欧美市场，对外贸易持续较快增长。2005年全市产品出口国家和地区发展到104个，比“九五”期末增加20个。积极拓展国际经济文化交流空间，与挪威王国拉尔维克市签订了关于友好合作关系的共识备忘录，与保加利亚韦林格勒市和斯洛伐克特伦钦地区签订了加强交流与合作备忘录；积极参加泛珠三角经济合作，与贵州省黔西南州缔结为友好市州，初步建立了我市的燃煤和劳动力补充基地；积极加强与国内外大型企业的合作，艾默生集团、伊斯曼集团等全球知名企业先后到我市进行实业投资，与中国材料集团、华润集团等的经济合作正在逐步开展；积极参与“山洽会”、粤港、粤台等经贸活动，2005年全市共签订经济合同、协议项目共88个，金额107.6亿元。

——交通、能源等基础设施建设取得重大突破。广梧高速公路一期马安至河口段工程于2004年底建成通车，二期河口至双凤段工程已动工建设，云浮至广西岑溪高速公路（粤境段）预可行性研究报告评审等前期工作基本完成。改造了国道324线云城西段，建成了新世纪大道；改造和新建了省道113、352线等一批县级以上公路以及镇通村公路，全市通车公路总里程达4435.8公里，比“九五”期末增加651.2公里。四围塘和都杨码头的规划和引资建设工作已全面启动。能源建设大步推进。云浮发电厂二期和罗定粤泷电厂两台机组相继建成投产，全市发电装机容量已达115万千瓦，比“九五”期末增长88.5%；投资8.59亿元，增加输变电设施，改造城乡电网，实现了城乡同网同价。建立了政府公众、政务、农村经济、企业信用等信息网站，电子口岸、财政结算管理、电子公文交换、视频会议、地税“大集中”工程等一批应用系统投入使用。建成市区生活污水处理厂等环保设施。投入水利建设资金12.01亿元，完成了5宗城乡水利防灾减灾工程和一批小型水库除险加固工程。

---规划、建设上新水平，城市品位不断提高。五年来，先后完善配套了市人民广场等公众活动场所，建成了市艺术馆、市电信综合大楼、市广电大厦、财政大楼、检察大楼、青少年活动中心等一批重点工程。共完成工程项目建设881项，总面积556万平方米，造价42亿元。市委党校、市图书馆、博物馆、档案馆等正在加快建设，城市立面改造、城市景观建设也有新的进步。发展了市区城市花园、新兴翔顺花园、罗定龙江花苑等一批城市住宅小区。中心城区通过扩建路网、实施美化绿化、增设城市景观，不断扩大城市规模和提高城市品位。各县（市）也对城区主干道和沿江公路进行了改造，城镇基础设施进一步完善，辐射和集聚功能逐步增强。完成了中心镇规划修编工作，加快了村镇建设步伐，“十五”期间全市共投入村镇建设资金27.62亿元，其中投入中心镇建设16.68亿元。全市城镇化水平达到43.04%，比“九五”期末提高了10.88个百分点。

——市、县、镇三级经济加快发展。市级经济实力逐步增强。省属企业规模扩大，产业链延长，城区第三产业蓬勃发展，市与县（区）合作共建工业园区开局良好。2005年，市级财政一般预算收入2.69亿元，比上年增长18.17 %，五年年均增长27.4%。县域经济发展加快。云城的石材、服装和第三产业，罗定的电子、纺织服装，新兴的不锈钢制品、农产品加工，郁南的机械、电池，云安的水泥、化工等特色产业规模不断扩大。农业产业化经营规模扩大、水平提高，覆盖了全市各县（市、区）。广东温氏集团等一批龙头企业已成为县域经济发展生力军。全市个体私营企业达到4.58万户,其中私营企业1585户，分别比“九五”期末增长62%和408%。2005年民营企业工业产值占全市工业总产值的88.5%。2005年，云安、郁南、新兴和罗定市的财政综合增长率分别排在全省67个县（市）第4位、第7位、第 9 位和第36位。镇域经济出现了加快发展的好势头。2005年，全市镇（街）新上投资500万元以上的项目122个，投资总额46亿元。63个镇（街）共实现税收5.6亿元，比上年增长34.6%，高于全市平均增长水平。其中税收1000万元以上的镇（街）11个，比上年增加3个。

——各项改革不断深化。围绕建设廉洁高效政府目标，我市不断深化各项改革。2001年开展的机构改革党政群机关行政编制总体精简20%；2003年完成撤并镇11个。2005年实现全免农业税。大力推进行政审批制度改革，审批项目减少了32.53%。设立市、县行政服务中心，提高审批效率和透明度。推进公共财政建设，开展部门预算、国库集中支付、财政支出绩效评价和政府采购等改革。加强对重点领域、重点部门、重点资金的审计监督，查出违规金额25488万元，上交财政金额2913万元。全面实施招投标制度，政府投资工程和土地、矿业权出让招投标率均达100%。基本完成了国有企业改革，改革面达95%。社会保障制度改革有新突破，参加养老保险人数达17.3万人，比“九五”期末增加了7.97万人。率先推行新型农村合作医疗制度，2005年覆盖率达82.9%，高出省定标准32.9个百分点。率先在公用事业服务项目中引入招标竞争机制。金融体制改革扎实推进，较好地化解了农村合作基金会、城市信用社金融风险，维护了广大群众的利益。完成文化、广播电视、新闻出版和粮食等体制改革。住房、投资、卫生、教育等各项改革工作进一步推进。

——各项社会事业和精神文明建设不断取得新发展。2005年，全市科技进步对经济增长贡献率达到48.5%，比“九五”期末增加8.5 个百分点。全市有省级工程中心8个，省级以上高新技术企业32家，省级技术创新专业镇12个，分别比“九五”期末增加8个、26个和12个；省级农业科技创新中心4家，民营科技园、农业科技园各1家;到2005年全市已有广东省名牌产品9个、全国免检产品3个、省著名商标9个，分别增加7个、3个和9个。建立了华南农业大学云浮产业化实验区，成立了华南理工大学云浮研究院。九年义务教育进一步巩固提高，“十五”期间，先后投入3.8亿元发展高中阶段教育，新建和扩建了云浮中学高中校区等6所高中校舍。完成了172所山区、老区薄弱学校改造工作。高考连年取得好成绩，2005年专科A线上线率比省平均水平高8.3个百分点。中小学校“一机两语”特色教育走在全省前列。创办了罗定职业技术学院，新组建了市技工学校，启动了市职业教育校区的筹建工作。“十五”期间共投入文化设施建设资金1.1亿元，提前一年完成省人大议案提出的任务。民间传统文化、特色文化、高雅文化相互交融，文化市场日益繁荣。成功举办了六祖文化节和南江民间文化艺术节，开展南江文化研究发掘工作，罗定市、新兴县被评为国家级文化先进县（市），郁南县连滩镇被命名为“中国民间艺术之乡”。《云浮市志》编修工作不断推进。全市广播电视光纤干线实现联网，建成大绀山广播电视发射台。实施体育“民心工程”，建成市健身广场等一批城乡体育设施。我市举重、摔跤、击剑、划艇、女子曲棍球等项目在国内外比赛中获得好成绩。参加世界赛获金、银、铜牌各1枚，参加亚洲赛获金牌2枚、银牌1枚；成功举办首届市残疾人运动会。完善了疾病预防控制、医疗救治、卫生监督三大体系，有效防控了非典型性肺炎、人禽流感、狂犬病等重大疫情。建设和改造镇卫生院42间，实现了省卫生村、镇零的突破。全面开展药品集中招标采购和医疗器械招标采购。药品集中招标采购让利群众8594万元，医疗器械招标采购比预算价减少734万元。卫生监督机构建设得到加强，强化药品食品卫生监督，严厉打击非法行医。2005年，我市被省授予创建农村中医工作先进市示范单位。实施了农村部分计划生育家庭奖励办法、“节育奖”和优惠制度，人口出生率、自然增长率连年完成省下达的指标。全面完成省下达农田保护任务，整顿和规范矿产资源开采秩序取得阶段性成效，地质灾害得到有效防治。全面开展创建省“双拥模范城”活动，部队建设、退役官兵安置、随军家属就业以及人防工程规划建设等工作得到加强。完成了“十一五”规划的编制、经济普查和1%人口抽样调查。深入开展邓小平理论、“三个代表”重要思想、科学发展观理论学习活动和“三讲”、保持共产党员先进性等思想教育活动；大力开展公民道德建设，建设了一批文明行业、文明单位、文明村镇和文明社区。

——民主和法制建设进一步加强。坚持和完善向人大及其常委会报告工作、向政协通报情况的制度，自觉接受监督。“十五”期间，市政府共出台规范性文件56件，办理人大议案、建议315件和政协提案394件，受理行政复议案件118件。加强与工会、共青团、妇联和工商联等群众团体和党外人士的联系，进一步拓宽民主渠道；完成了城市、农村基层体制改革和民主选举村民委员会、社区居委会工作。大力整顿和规范市场经济秩序，禁止行业垄断和地区封锁。建立了公开交易经营性用地、采矿权有偿出让和政府采购招投标制度。加强领导干部任期经济责任审计。深入开展法制宣传教育，圆满完成“四五”普法工作任务。加强人民调解工作。认真贯彻落实《国家公务员暂行条例》、《行政许可法》等法律、法规和规章，加大力度推进政务公开，先后两次开展“万人评公务”活动，机关工作作风明显改善，政务环境进一步优化。

——和谐云浮建设扎实推进。各项民心工程成效显著。全市低保实现了应保尽保。2005年发放低保资金1764.58万元，受惠群众5.89万人，分别比“九五”期末增长4.6倍和4.9倍。五年内共投入救灾资金4115.5万元，受惠群众49.81万人次。社会保险覆盖面进一步扩大，2005年实收社保费36752万元，比“九五”期末增长159.27%。全面落实就业和再就业政策，采取多种综合措施促进劳务经济发展，五年来，全市新增就业岗位13.26万个，农村劳动力转移就业18.15万人。城镇登记失业率稳定在较低水平。广泛开展智力扶贫工程。农村新型合作医疗快速发展，初步建立起农村医疗保障体系。在全省率先解决了18万农民的饮水难问题，去年重建了523户西江特大洪水毁坏的灾民房屋。建成了413条生态文明村，受惠群众达13.8万人。动工改造城乡贫困户危房3140户。建成106户特困单亲母亲家庭安居房。对拖欠农民工工资、拖欠征地款和建设领域拖欠工程款等进行专项清理，取得较好成效。共办理法律援助案件2736件，有效解决群众打官司难问题。切实加强社会治安综合治理，组织开展打击“双抢”、“双盗”和侦破命案等专项行动，严厉打击“六合彩”、“法轮功”邪教和非法传销等各种违法活动，实行信访工作目标责任管理，坚持领导接访和领导包案调处信访大要案制度，切实抓好集中处理信访案件和解决信访突出问题工作，及时化解各类社会矛盾。落实安全生产责任制，强化领导责任，深化安全生产专项整治，安全事故明显下降。环境保护和生态屏障建设得到加强，西江、新兴江、南江和南山河水环境综合整治工程和重点污染源治理工程扎实推进，城区降尘量从2003年起连续三年控制在省的标准以内。创建林业生态市工作完成阶段性任务，郁南、罗定被省政府授予广东省林业生态县（市）称号。森林资源保护得到加强，生态环境进一步优化。

此外，物价、编制、知识产权、经济发展研究、民族宗教、档案、武装、国防动员、人民防空、民兵预备役、外事侨务、台务、消防、保密、新闻出版、贸促会、信息产业、气象、侨联、工商联、文联、社科联、妇女儿童、红十字会以及驻外机构等也取得了新的成绩。

各位代表, “十五”计划的胜利完成，使我市的综合实力进一步提高，经济和社会发展的基础更加扎实，为我市“十一五”和未来一段时期的加快发展奠定了良好基础,标志着山区经济强市建设迈进新的发展阶段。这是在党中央、国务院，省委、省政府和市委的正确领导下，全市人民奋发向上，积极进取的结果。在此，我代表市人民政府，向全市广大工人、农民、知识分子、干部职工，驻云浮解放军、武警部队、消防官兵和公安干警及各人民团体、社会各界人士表示崇高的敬意！向关心和支持云浮建设和发展的香港、澳门特别行政区同胞、台湾同胞和海外侨胞及国际友人表示衷心的感谢！

回顾“十五”时期的工作，我们深深体会到，要实现我市国民经济和社会各项事业的加快发展，必须始终坚持以邓小平理论和“三个代表”重要思想为指导，认真落实科学发展观，求真务实，开拓进取，狠抓落实,结合实际创造性地开展工作。一是要把加快经济发展作为中心任务，抢抓发展机遇,尤其是世界范围内出现的产业转移的机遇。二是要加快工业化进程，抓住工业化这个龙头，带动城镇化和农业产业化发展，不断优化全市经济结构。三是要调动各方积极性，形成加快发展合力，促进市、县、镇经济全面发展，内源、外源经济一起上，以壮大全市经济总量。四是要加大招商引资力度,加快工业园区建设步伐,狠抓项目建设工作的落实，增强工业经济发展后劲。五是要坚持城乡协调发展，加快农业和农村经济发展，不断改善农村的生产和生活环境，切实解决好“三农”问题。六是要注重全市大局稳定，及时化解各种社会矛盾和群众纠纷，坚决打击各种犯罪活动，为实现经济社会加快发展协调发展提供良好的社会环境。

同时，我们也清醒看到，当前我市经济社会发展仍存在不少制约因素和薄弱环节，主要是:经济总量小，工业化程度低，政府财力较弱；工业园区建设还不能满足工业发展需要；外贸出口和利用外资规模小，对经济发展拉动力不足;农民收入增长缓慢，持续增收难度较大；农村尤其是石灰岩苦旱镇生产生活条件改善任务繁重，改造农村水利和交通设施的工作还很艰巨；各项社会事业发展的投入不足，发展缓慢；与人民群众切身利益密切相关的安全生产、社会治安、医疗卫生、食品安全等问题依然突出。调整经济结构、增强自主创新能力、优化经济发展软硬环境等方面还有大量的工作要做。对此，市政府将在今后工作中，采取切实有力措施，认真加以解决。

“十一五”时期的奋斗目标和主要任务

“十一五”时期，是我市建设山区经济强市、全面建设小康社会的关键时期。我市既面临难得机遇，又面临严峻挑战。当前经济全球化和区域一体化呈加快发展趋势，大大拓展了我国经济发展空间，我国经济正处于新一轮增长周期的上升期；我省经济自主增长的动力不断增强，与港澳及泛珠三角地区各省的经贸合作关系更加紧密；我市经过“十五”期间的发展，积累了一定的产业基础、经济实力和成功经验，发展思路清晰，方向明确，措施具体，具备了加快发展的基础条件。但与此同时，我市与珠三角等发达地区的发展差距大，全省各地尤其是山区市的加速发展，使我市面临很大的竞争压力、挑战压力和赶超压力。为此，我们一定要解放思想，创新观念，把握好新的发展机遇，以加快发展作为全市各级的中心工作，以加快发展破解各种难题，围绕加快发展狠抓工作落实，努力实现建设山区经济强市、构建和谐云浮的宏伟目标。

根据中共云浮市委关于“十一五”规划的建议，市政府组织编制了《云浮市国民经济和社会发展第十一个五年规划纲要》，提出了“十一五”期间，全市生产总值年均增长12%以上，单位生产总值能耗比“十五”期末降低15%以上的发展目标。

实施“十一五”规划，重要的就是坚持以邓小平理论和“三个代表”重要思想为指导，把科学发展观贯穿到推进我市改革开放和现代化建设的全过程，贯穿到经济和社会发展的各个方面。要始终坚持“六个必须”：一是必须加快经济发展。要积极推进工业化、城镇化和农业产业化，做大经济总量，做强政府财力。二是必须优化发展环境。要不断优化投资软环境，改善投资硬环境，吸引更多的企业投资云浮、扎根云浮，共同发展。三是必须加快转变经济增长方式。继续调整和优化产业结构，坚持节约发展、清洁发展、安全发展，实现可持续发展。四是必须提高科技创新能力。不断推进我市产业改造升级，提升产业和经济竞争力。五是必须加快建设社会主义新农村。贯彻工业反哺农业、城市支持农村的方针，采取有力措施逐步改变农村的落后面貌，促进城乡协调发展。六是必须加快和谐云浮建设。把构建和谐云浮，全面建设小康社会作为加快发展的目的，使全市人民过上更加宽裕、更加安宁、更加幸福的小康生活。在加快发展过程中，要十分注重提高经济增长的质量和效益，十分注重资源节约和环境保护，十分注重把改革的力度、发展的速度和社会可承受的程度统一起来，在社会稳定中推进改革与发展，通过改革和发展促进社会稳定。

为实现“十一五”规划目标任务，必须做好八方面工作：

（一）加快工业化进程，壮大经济总量。一是发展壮大外源型经济。要充分利用国际国内两个市场、两种资源，充分利用“山洽会”和省组织的外经贸招商活动平台，以及省委、省政府实施的山区市与珠三角联手推进产业转移等政策，加大对外招商，积极承接珠三角产业转移。二是做大做强民营经济。认真落实好省、市的一系列加快民营经济发展的政策措施，积极扶持民营经济，培育发展主业突出、市场竞争力强的大企业和企业集团。三是加强工业园区建设。重点抓好都杨、新成工业园区和思劳高新技术产业园的规划、开发和建设，抓好各县（市、区）工业园区建设。四是拓展区域合作空间。继续推进与佛山市等珠三角地区的经贸合作，加快融入珠三角经济圈，加强与黔西南州等泛珠三角地区的区域合作。开发利用境外资源，开拓海外市场，扩大我市对外经济规模。

（二）着力解决“三农”问题，努力建设社会主义新农村。一是继续提高农业综合生产能力。继续推进农业产业化经营和农业科技进步，加强农业基础设施建设；增加农业投入，抓好粮食生产，发展林业产业，加快农产品流通和加工业发展。二是深化农村综合配套改革。稳定并完善以家庭承包经营为基础、统分结合的双层经营体制；三是建立完善农村公共事业体系。加快建立城乡平等就业制度和统一的劳动力市场，全面推进城乡统筹就业。大力实施“智力扶贫”和“百万农村青年技能培训”工程，发展远程教育和广播电视“村村通”，完善农村通讯基础网络建设，全面改善农村卫生保健、医疗救治条件。四是推进新农村建设。改善农村生产生活条件，倡导文明、健康的生活方式，改革农村社会管理方式，提高村民自治水平。五是努力增加农民收入。在大力发展农业生产的同时，加快发展劳务经济，增加非农收入，整村推进扶贫开发，加快贫困群众脱贫致富奔康步伐。

（三）提高综合竞争能力，促进区域协调发展。一要规划建设合理分工的区域城镇体系，构建组合有序、优势互补、整体协调、持续发展的城镇体系。二要提高城镇化的水平和质量。引导人口、资金、信息等各种生产要素向城镇和工业园区集聚，促进城镇化与工业化的互动。三要加强基础设施建设。突出抓好高速公路建设、省道368线六都至高要大湾段扩建改造和通镇、通村公路硬底化，抓好罗岑铁路建设和都杨码头等港口码头综合开发。积极争取云浮电厂三期、华润西江发电厂、内河核电等能源项目立项建设，加快城乡电网改造和城乡防灾减灾体系建设。四要促进市直、县域、镇域经济全面协调发展。加快发展市级工业和中心城区第三产业。以招商引资和大力发展民营经济为主要途径，发展壮大县域、镇域经济，不断增强县、镇经济实力。

（四）坚持科技引进与自主创新相结合，推进产业结构优化升级。一是建立和完善科技创新体系。加强与高等院校、科研院所的经济技术合作，支持企业建立各种形式的研发机构，大力培育高新技术企业。二是运用高新技术改造传统产业。积极鼓励现有企业增加科技投入，增加产品的高新技术含量。到2010年，高新技术产品产值和增加值分别占全市工业总产值、工业增加值的20%以上和30%左右。三是发展先进制造业。引导资源向优势产业集中，发展企业集群，做大做强支柱产业，打造一批名牌产品、著名商标和知名企业。重点发展电子机械、精细化工（硫化工）、新型材料、生物制药、新能源及环保等具有一定先进水平的制造业。四是提升服务业发展水平。积极推进创建中国优秀旅游城市活动，发展壮大旅游业，加快发展房地产、商贸流通、交通运输、仓储和物流配送等服务业，培育发展信息、金融、保险等现代服务业，鼓励发展法律、会计、咨询、评估等中介服务业。

(五)加大体制改革和创新力度，增强经济社会发展活力。一是推进政府管理创新。进一步建立和完善各类激励机制，综合运用行政和经济手段，调动公务员抓落实的积极性。建立健全依法、科学、民主决策机制，加快建设服务型、依法行政和廉洁高效政府。二是加强国有资产监督管理。完善公司法人治理结构，强化企业财务监督。加强政府资源的整合、统筹、开发与利用，深化垄断行业改革与监管。三是完善财政体制改革，稳妥推进地方金融体制改革。积极稳妥地推进农村信用社改革，支持商业保险实行公平竞争。四是建立健全现代市场体系。规范产权、资本、土地、劳动力、技术等要素市场，建立健全社会信用体系。

（六）加快发展文化和社会各项事业，全面提高城乡文明水平。一是深入开展现代公民教育活动，提高广大干部群众的政治理论、思想道德和文化水平。二是加强教育和人才工作。统筹城乡教育发展，增加资源投入，建立农村义务教育经费保障机制，巩固九年制义务教育。加快发展高中阶段教育、中等职业技术教育、高等职业教育、学前教育、成人教育。要切实重视人才培养与引进工作。三是推动文化体育工作上新水平。深入开展群众文化活动，健全和发展文化市场，整合文化资源、打造文化精品，加快有线电视网络改造步伐。完善体育设施建设，积极开展体育竞技和全民健身活动。四是加快发展医疗卫生事业。深化医疗卫生改革，巩固完善公共卫生三大体系建设，加强城市社区和农村卫生工作，巩固和提高农村合作医疗水平，加快中医药事业发展。

（七）推进可持续发展，努力构建和谐云浮。一是继续做好人口与计划生育工作。二是加强资源节约和环境保护。三是积极解决社会就业，提高社会保障水平。四是保持居民收入持续增长。五是维护好社会稳定。

（八）全面推进民主法制建设，不断提高社会管理水平。一是大力推进依法治市，全面贯彻实施《行政许可法》和《实施纲要》，建设法治政府。二是加强民主政治建设。坚持和完善各项民主政治制度和政策，支持各人民团体依法依章开展工作，加强基层民主政治建设。三是加强社会管理。加强社区建设，增加公共服务投入，提高服务水平。

各位代表，展望我市“十一五”的发展前景，我们充满信心。我们坚信，只要我们进一步增强使命感、紧迫感和责任感，埋头苦干，狠抓落实，就一定能够实现我市“十一五”经济社会发展各项目标，建设一个生产发展、生活安康、环境优美、生机勃勃的新云浮。

2006年工作目标任务和总体要求

各位代表， 2006年是我市实施“十一五”规划的第一年。政府工作的总体要求是：高举邓小平理论和“三个代表”重要思想伟大旗帜，进一步树立和落实科学发展观，认真贯彻中央经济工作会议、省委九届八次全会精神，积极实施“东渐西联、优内拓外、错位竞争、统筹并进”发展战略；加快工业发展步伐，带动城镇化、农业产业化，优化经济结构；加大招商引资力度，加快项目建设进度，促进市、县、镇三级经济发展；加快发展科技、教育、文化、卫生、体育等各项事业，促进全市经济社会协调发展；坚持依法治市、依法行政，加强政治文明和精神文明建设；坚持以人为本，注重社会公平，加强利益协调，构建和谐云浮，为实现建设山区经济强市和全面小康社会宏伟目标打下坚实基础。

根据上述要求，2006年全市经济和社会发展的预期目标是：生产总值增长13.5%，其中第一产业增长5.2%、第二产业增长23%、第三产业增长13%，单位生产总值能耗下降3%，社会消费品零售总额增长15%，全社会固定资产投资增长25%，地方财政一般预算收入增长18%，外贸出口总值增长15%，实际利用外资增长30%，农村居民人均纯收入增长5%，城镇居民人均可支配收入增长12%，人口自然增长率控制在7‰以下，城镇登记失业率控制在3.4%以内,居民消费价格总指数控制在3%以内。

实现2006年的目标，我们的有利条件较多。一是当前我国宏观经济仍保持较快速度增长，产品销售畅旺。我市工业、农业和第三产业仍在良好的宏观经济形势下发展。二是省委、省政府进一步加大对山区市发展的扶持力度，加大对山区交通、水利等基础设施建设的支持，有利我市发展环境的改善；积极推动珠三角地区产业向山区市转移，有利我市工业加快发展。三是通过调动市、县、镇各级各部门的积极性抓落实促发展，2005年我市部分经济指标在省内地级以上市排位逐步上升，社会事业发展速度加快，一些工作排在全省前列，实现加快发展已有较好的工作基础。四是我市经济社会已步入了加快发展的轨道，高速公路等基础设施项目加快推进，一批引进项目在建、申报立项和在谈，我市经济社会新一轮加快发展的格局已初步形成。只要我们进一步增强使命感、紧迫感和责任感，埋头苦干，狠抓落实，就一定能够实现2006年经济社会发展预期目标任务。

实现今年经济社会发展预期目标，必须做好以下十方面工作：

（一）加快工业发展步伐，壮大全市工业规模。

走新型工业化道路，加快工业经济发展步伐是我市经济工作重点。要继续做大做强现有几大工业产业，发展产业集群。同时，今年要努力在工业园区建设、招商引资、上大项目等方面有新突破，以推动全市工业加快发展。

要努力提升现有工业产业的发展水平。引导石材、不锈钢制品、硫化工、水泥、纺织服装、机电、制药及农产品深加工等行业增资扩产，努力培育工业龙头企业，开发和生产系列产品，延伸产业链，带动上下游配套产业发展。大力发展产业集群。充分发挥行业协会组织的作用，加强企业生产、贸易、技术合作，实现优势互补、集约发展。要积极运用先进适用技术和现代管理方式，改进设备和工艺技术，增加产品技术含量，提高企业管理水平。要努力提高企业自主创新能力，积极鼓励和帮助企业创名牌。积极支持省驻云浮国有企业投资新项目，扩大生产，提高经济效益。

大力推进项目建设，努力实现工业生产增量扩张。抓好云浮电厂三期、华润西江发电厂、内河核电的申报立项和筹建工作，继续做好粤泷电厂二期、抽水蓄能电站等大项目的有关论证工作。切实落实项目工作责任制，进一步完善重点项目抓落实的台帐管理制度，加快在建项目建设进度，尽快形成生产能力。加强已签约项目的跟踪落实，提高合同履约率。继续加大项目的引进力度，争取年内引进一批骨干项目，以增强工业发展后劲。

加快工业园区建设，积极主动承接珠三角地区产业向我市转移。工业园区要坚持高起点规划、高标准建设、高效能管理，节约集约用地，创新筹资机制，加快建设步伐。重点推进都杨、新成产业转移工业园区和思劳高新区的规划、建设，各县（市、区）也要加快建设一批工业园区，扩大承接转移企业能力。制订好企业进园区的各项具体优惠政策，加快完善园区内水、电、路、环保和信息化等配套设施建设，加大园区招商推介力度，促进项目早进园区。

切实加强对工业发展的领导和服务工作。做好工业产业发展规划及结构调整，加强对工业发展形势的研究，注重引导工业企业做大做强和优化工业产业结构。加强对工业经济的服务和协调，注重协调解决企业生产中煤、电、油、运、用地和用工等困难。加快资本市场的开发利用，加强对企业改制上市工作指导。用好国家的金融政策，发挥好中小企业担保机构效能，积极开展与市内外金融机构的合作，争取金融机构支持，解决企业融资难问题。积极与高等院校、科研院所合作，为工业发展提供科技动力和智力保障。加强企业用工培训。政府各部门要当好创造发展环境的主体，努力提高为企业服务的工作效率和质量，为工业发展提供高水平服务。

（二）以农业产业化为龙头,加快农村经济发展,推进社会主义新农村建设。

今年我市农业农村工作，要在继续抓好粮食生产基础上，继续推进农业产业化经营，发展好特色农业和劳务经济，增加农民收入，努力改善农村生产生活条件，推进农村基层民主管理，努力在建设社会主义新农村方面取得新成效。

进一步推进农业产业化经营,扩大覆盖率，提高农业生产效益。推广温氏模式和经验，引导农业龙头企业实行股份制经营，增强发展活力，促进龙头企业做大做强；引导龙头企业和农业商品生产基地相结合，发展集约农业、规模农业和高效农业；引导农村经济合作组织、农业龙头企业利用我市资源优势大力发展种养业、农产品深加工以及流通业、资源深加工及相关产业；继续引导龙头企业到各个石灰岩苦旱镇发展，带动农民致富。建设农业现代化示范区，提高农业资源开发水平和效益。

大力发展特色农业，打造我市农业品牌，确保实现农业增效。依托各地农林资源，加强农业招商引资。继续发展云城的蔬菜、花卉,罗定的松脂、肉桂,新兴的花卉、优质稻，郁南的无核黄皮、沙糖桔，云安的蚕桑、柑桔等农业商品生产基地，加强农产品商标和地理标志的培育创建力度。增加农业科技投入，提高农产品科技含量和质量，发展绿色食品。发挥现有农贸专业市场的集散功能，积极吸引商家和农户入场经营，搞活农产品流通；加快粤西泷州农产品交易中心二期和思劳农产品交易市场建设。积极发展罗非鱼等水产养殖业。扩大南药种植，发展林业产业、城市绿化苗木培植和林产品深加工等，提高林业经济效益。继续推进林业生态县建设。

加快发展劳务经济，增加非农收入。全面实施 “百万农村青年技能培训”工程，引导农村富余劳动力向非农产业和城镇转移就业；继续实施“智力扶贫”，使贫困地区群众有一技之长，增强自我发展和到非农产业就业能力；发挥本地企业培训效能，推广订单式培训，重点培训电子、纺织服装、石材、不锈钢制品、水泥等行业和各类服务业的用工。今年政府将拨出专项资金，培训农民工1.6万人。

统筹开展建设社会主义新农村工作。加强基本农田保护和农业基础设施建设，认真搞好农业综合开发，积极配合省开展新农村建设试点和“百镇万亩、千村千亩改低示范行动”计划等工作。今年计划改造中、低产田4.3万亩。加快实施农村安居工程，今年完成3500户贫困户危房改造计划，确保2007年前基本完成农村贫困户危房改造任务。抓好高致病性禽流感等重大动植物疫病的防控工作。积极培育、繁荣和规范农村市场，加大对农村市场商品质量监管。深化农村综合配套改革，全面落实免征农业税、种粮补贴、农机补贴、粮食最低收购价等各项政策。加强社会主义新农村建设工作的统筹规划，今年继续安排建设生态文明村600条，加快发展农村沼气，开展创建卫生村活动，努力改善农村生产生活条件。按照生产发展、生活宽裕、乡风文明、村容整洁、管理民主的要求，加快社会主义新农村建设步伐。

（三）继续加强交通、城建、水利和环保等基础设施建设，努力改善经济发展环境条件。

以加快高速公路建设为重点，推进交通网络建设。积极配合业主单位做好云梧高速公路建设施工的各项服务工作；加快云浮至广西岑溪高速公路（粤境段）申报立项等前期工作。努力争取上级的支持，把揭阳至茂名高速公路（思劳至新兴段）等纳入规划，并早日启动建设。今年计划投入公路建设资金15.05亿元，其中高速公路完成投资12亿元，同时投入3.05亿元建设改造588.8公里国省道、通镇通村公路。加快云安六都至高要大湾沿江公路建设项目的申报立项工作，争取尽快动工建设。加快四围塘码头的建设进度以及都杨码头的招商筹建。继续推进罗定至岑溪段铁路筹建工作。

推进城镇化建设，增强对经济和社会发展的综合服务功能及辐射能力。加快市城区道路改造步伐，重点开展兴云中路、兴云西路、城中路、城南路、建设路、河滨路等城区主要道路改造，开通河滨南路凤凰桥至金华桥段、金山大道兴云西路至邓发纪念中学路段。编制公共交通规划，积极发展城市公共交通服务。继续推进市委党校、市迎宾馆三期、市图书馆、博物馆、档案馆和市老干部活动中心等项目建设。支持省驻云浮单位各项建设工程加快建设进度。加快修编市区总体规划及“十一五”近期建设规划，完善县城和中心镇控制性详细规划，加快供水、供电、环保和卫生等市政公共项目建设。继续推进市城区、县城和中心镇住宅小区建设。加强社区管理，完善城镇功能，优化居住环境，引导农村人口向城镇集聚，加快城镇化进程。

切实抓好水利和公共卫生设施建设。市区南山河防、郁南县城防等水利工程，要争取在年内全面动工，完成5宗城乡水利防灾减灾工程、23宗小型水库除险加固等工程，解决15条水库移民村的遗留问题。从今年开始，启动城乡群众饮水安全工程建设，分期分批解决全市121万群众的饮水安全问题。加快市区生活垃圾卫生填埋场等8 个环保项目建设，年内要建成4个。

（四）走“三化”路子，大力推进县域经济发展。

加快工业化、城镇化、农业产业化发展步伐，促进县域经济发展。以工业化为龙头，以民营经济为主体，培育县域优势产业、优势企业和优势产品，各县（市、区）要围绕做大做强石材、硫化工、不锈钢制品、水泥、纺织服装、电子电器、农产品深加工等工业支柱产业，主动“走出去”开展招商引资，大力吸纳珠三角产业转移。加快环保工业园区建设。认真落实镇（街）引进项目到市内异地兴办企业的利益分成政策，支持偏远镇开展招商引资工作。

加快第三产业和民营经济发展。继续推进县城、中心镇房地产业发展，发展壮大商贸流通、餐饮、交通运输、旅游、信息、金融等各类服务业。充分利用自然生态、人文历史等资源条件发展旅游业，积极整合旅游资源，提升旅游景点档次和接待水平。开展创建中国优秀旅游城市活动，争取2007年通过国家旅游局验收。落实好国家和省、市加快民营经济发展的优惠措施，放宽准入条件和领域；引导和支持符合条件的民营企业创新经营机制实行股份制改造，推动民营企业资本合作，逐步培育发展主业突出、市场竞争力强的民营企业集团。完善民营经济服务体系，各部门都要营造为民营企业服务的良好氛围，积极为民营企业做好用地、用水、用电、招工、人才引进、科技引进、创立品牌等服务工作。加快发展中小企业信用担保业务。

继续大力推进镇域经济发展。落实省关于加快山区县、中心镇、专业镇发展等有关政策和措施，继续实施镇域经济工作责任考核制度，用经济和行政的激励措施，调动镇（街）领导干部发展镇域经济的积极性。继续推进农村工业化，大力开展招商引资，积极引进各类资源加工和劳动密集型企业，使农村工业的发展与本地资源开发利用、农林牧渔产业发展以及农民的增收紧密地结合起来。要宜工则工、宜农则农、宜牧则牧，区位条件或产业基础较差的镇，可以先引进农业龙头企业发展种养业和相关加工业，逐步带动其他行业加快发展。加快城镇基础设施和公共设施建设，优化发展环境，实现全市镇域经济加快发展。

（五）进一步加强外经贸工作，做大外源型经济。

抓住世界范围内产业转移有利时机，大力度多形式多层次开展招商引资工作，力争今年招商引资工作又有新突破。今年要认真组织好第三届中国（云浮）国际石材科技展览会，积极参加省组织的粤港、中东、欧洲等经贸活动。要特别重视做好世界级大企业、名牌企业的招商和引进工作；同时要重视引进大批量的中小项目。充分利用招商网络和驻珠三角招商联络处等平台，抓住珠三角产业转移机会，实施优惠措施，大力吸引珠三角产业转移项目到我市落户。充分利用我市的产业和资源优势，注重项目和园区的宣传及包装推介工作，增强推介招商效果。创新招商形式，拓宽引资渠道，促进招商引资企业化、专业化、社会化，充分发挥政协委员、海外社团和行业协会（商会）等的桥梁作用，大力推行以商引商，积极做好外资企业服务工作，鼓励外资企业增资扩产。完善招商引资工作层级责任制和考核奖惩制度，加强对招商引资项目的协调服务，提高项目引进工作成效。

扩大我市产品出口规模。积极落实国家调整后的出口退税政策，放宽出口企业代理权等方面的政策限制，为出口企业提供更多的信息、法律和中介等服务，鼓励和支持民营企业获取出口经营权，扩大出口。实施科技兴贸，优化出口商品结构，扩大具有自有知识产权、自有品牌和高新技术产品、机电产品，以及扶持农产品深加工出口，提升纺织服装、不锈钢制品、石材等产品出口档次。抓紧“云浮出入境检验检疫局”的筹建，加快建立石材和不锈钢两个保税仓，优化口岸服务工作。在巩固港澳台、东南亚、欧美日等传统市场的同时，积极开拓非洲、南美、俄罗斯和中东等新市场，引导和支持有条件的企业到境外开设分支机构，拓展发展空间，扩大对外合作。

（六）完善财政管理体制，进一步强化财税工作。

大力培植财源。加快二、三产业发展和重点项目建设，继续推进政府资源的开发和管理，形成支撑有力、后续旺盛、结构合理的财源体系。努力打造依法诚信纳税环境，严格依法治税，着力提高税收征管的质量和效率，提高征管水平。进一步加强和规范非税收入管理，确保财政收入持续稳定增长。

优化支出结构，确保重点支出。按照建立“以人为本”的公共财政支出体系要求，进一步调整优化财政支出结构。加大财政对农业、科技、教育、文化、公共卫生、重点项目和就业、社保等方面的投入。

强化监管，规范运作。积极推进国库集中支付改革，加强 “收支两条线”管理，积极稳妥推进财政支出绩效评价，进一步提高部门预算合理性。控制财政举债规模，防范财政风险。完善政府采购制度，落实增收节支的各项措施，提高财政资金的使用效率。建立健全与公共财政体制相适应的行政事业单位资产管理体制。

加强审计监督，确保国有资金依法合理使用。继续深化预算执行审计，涉及人民群众切身利益资金的审计，以及开展效益审计，提高财政资金使用效益。

(七)加强资源节约和环保工作，发展循环经济，建设节约型社会。

以电力、水泥、化工、石材、冶炼、不锈钢制品等耗能行业为重点，大力推行清洁生产、节能降耗和资源综合循环利用。支持创建循环经济示范县和环保工业园。加快循环经济相关技术的研发,推广应用新技术、新工艺、新设备和新材料，逐步淘汰能耗高、效率低、污染严重的技术和设备。

加强环境保护和生态建设。继续加强西江、南江河、新兴江、南山河综合治理，加强农村面源污染治理，切实保护饮用水源。严格执行建设项目的环保准入制度，加快建立污染源信息动态管理系统和重点企业环保信用档案。加强环保监督和执法，严厉查处违法排污行为。加强森林资源保护，加快生态公益林体系建设，切实纠正因发展经济林而破坏生态林建设行为，继续开展绿色城镇、绿色学校、绿色社区、生态示范区等绿色创建活动，营造绿色生态屏障。实施矿山地质环境恢复治理工作。加强地质灾害调查、监测、防治和防震减灾工作，提高气象预警预报能力。

在生产、建设和生活等领域广泛开展节能、节水、节材、节地等资源节约活动。广泛宣传和动员全社会共同参与节约型机关、企业、学校、社区创建活动，强化节约意识、节约观念，倡导节约文化、节约之风，让广大市民养成节约习惯和节约行为。政府各部门、企事业单位要增强节约观念，艰苦奋斗，勤俭办事，带动和促进建设节约型社会。

（八）努力解决好与人民群众切身利益密切相关问题，推进和谐云浮建设。

加强劳动保障工作。加快经济发展，促进社会就业，大力发展劳动密集型产业、中小企业和服务业，扩大城乡就业门路，今年要努力创造3万个就业岗位；落实再就业政策，帮助国有企业下岗人员实现再就业；统筹做好大中专学校、职业技术学校毕业生和复退军人以及城乡新增劳动力转移就业工作。加大劳动监察力度，规范企业用工行为，坚决制止和查处拖欠工资、侵犯劳动者权益等行为。继续抓好工程款清欠工作。加快城乡社会保障体系建设，完善城乡特殊困难群众的社会救助体系。加强住房公积金管理，抓好廉租住房建设，逐步解决全市城镇住房困难户住房。继续扩大社保覆盖面，加强保费征收和管理，确保养老统筹金当期收支平衡和社保待遇按时足额发放。健全对被征地农民的合理补偿和安置机制，认真解决失地农民的就业和生活出路问题。进一步巩固和推进新型农村合作医疗工作，加快农村药品监督网和供应网建设，认真落实推行农村免费义务教育政策，加快农村通村公路建设步伐，努力解决农村群众“看病贵”、“读书难”和“行路难”问题。加强妇女儿童、老年人、残疾人权益保障和救灾救济工作。

落实安全生产责任制，严格执行安全生产许可制度，从源头上防范各类生产安全事故；加大投入改善安全生产条件，强化企业安全主体责任，强化安全生产执法，加大对非煤矿山、危险化学品、烟花爆竹、消防、道路交通、建筑施工等领域的安全监管工作力度，用强有力措施遏制重特大安全事故发生。继续深入整顿和规范市场经济秩序。加快社会信用体系建设，严惩制假售假、商业欺诈、走私贩私、偷逃骗税、金融诈骗和侵犯知识产权等行为。严厉打击商业贿赂以及传销和变相传销活动。开展食品安全专项整治，让人民群众吃上安全、放心食品。

加强社会治安综合治理，维护社会稳定。严厉打击 “双抢”、“双盗”等多发性侵财犯罪、有组织犯罪、毒品犯罪以及严重暴力犯罪。加强娱乐场所管理，深入推进城市社区和农村治安防范工作，营造良好治安环境。深入贯彻实施《信访条例》，完善领导信访接待和包案责任等制度，切实解决群众信访反映的热点难点问题，有效预防和处置群体性突发事件。

（九）全面发展各项社会事业，加强精神文明建设。

继续推进社会主义思想道德建设。加强社会公德、职业道德和家庭美德教育。深入进行“爱国、守法、诚信、知礼”现代公民教育。深入开展群众性精神文明创建活动，进一步提高公民的文明水平。

加快发展科技事业，加强科技创新体系建设。积极运用先进适用技术改造我市传统产业。继续加强与高等院校和科研院所合作，加快建材、机械设备、农产品深加工、禽畜和水产养殖等科研成果的转化。加大科普力度，提高全社会科技意识。扎实抓好基础教育，大力发展高中和职业教育，继续抓好2所在建高中和4所职校扩建工作，启动市职业技术学校建设工作。继续深化“一机两语”教育，全面推进素质教育。要努力营造良好的干事创业环境，加强人才的引进和培训工作。加强农村基层文化建设，全面构建公共文化服务体系；繁荣文化事业，加强文化市场管理，促进文化产业发展。整合文化资源，加大文化遗产的保护力度；实施有线电视数字化转换工作，扩大广播电视无线发射覆盖面；完善疾病预防控制、卫生监督执法和医疗救治三大体系建设；推进医疗体制改革，规范医疗服务项目和收费，规范药品和医疗器械集中招标采购行为；加强疫情监测，严控人禽流感、非典型性肺炎、狂犬病和爱滋病等重大疫病发生。加强农村卫生工作，加快发展中医药事业，积极开展卫生村创建活动和红十字会体制改革。深入开展全民健身活动，做好省“十二运”备战参赛工作。坚持完善人口和计划生育目标管理责任和利益导向机制，综合治理出生人口性别失衡，开展创建计生优质服务先进县活动。支持和加强国防建设，推进创建省双拥模范城工作。做好民族、宗教、外事、侨务、台务、统计、民政、妇儿、气象、人防、档案、方志等工作。

（十）以提高行政能力为重点，建设人民满意政府。

努力建设法制型、服务型政府，切实提高政府行政能力和行政效能。

加强民主法制建设。坚持向人大及其常委会报告工作，自觉接受人大及其常委会的监督，认真落实人大及其常委会的决议、决定。主动向人民政协通报情况，接受民主监督。认真办好人大代表议案、建议和政协提案。广泛听取各民主党派、工商联、无党派人士的意见。充分发挥工会、共青团、妇联、侨联、红十字会等群团组织的桥梁纽带作用。坚持和完善基层群众自治组织、企事业单位的民主管理制度，坚持和完善政务公开、厂务公开，实施“活力民主，阳光村务”工程。

全面履行政府职能，提高经济社会管理能力。加快政府职能转变，全面履行经济调节、市场监管、社会管理和公共服务职能。深化行政审批制度改革，规范行政审批程序，增强政府工作透明度。加快电子政务建设，提高行政效率。建立健全各种突发事件应急机制，提高保障公共安全和处理突发事件的能力。

加强政府法制工作，提高依法行政能力。完善政府有关规章制度，坚持依法决策、依法办事、依法处理问题。加强和改进行政执法工作，强化行政执法监督，严格实行执法责任制和执法过错追究制，切实做到有权必有责、用权受监督、侵权须赔偿、违法要追究。

全面贯彻实施《公务员法》，加强政风建设，提高公务员队伍素质。切实加强和改善公务员教育和管理，进一步改进机关作风。大兴求真务实之风，狠抓各项决策和工作部署的落实，多为群众办实事办好事。积极参与以“三个走在前面”为主题的“排头兵”实践活动。深入开展廉政建设和反腐败斗争，严肃查处损害群众利益行为。努力建设政治坚定、业务精通、清正廉洁、作风优良的公务员队伍。

同志们，今年是实施“十一五”规划的开局之年，我市的发展和稳定工作任务繁重、责任重大。让我们高举邓小平理论和“三个代表”重要思想伟大旗帜，更加紧密地团结在以胡锦涛同志为总书记的党中央周围，全面落实科学发展观，振奋精神，齐心协力，扎实工作，为加快我市经济和社会发展，建设山区经济强市、构建和谐云浮而努力奋斗！
